# Supplementary material for: Community-intrinsic properties enhance keratin degradation from bacterial consortia
Source: PLoS One. 2020 Jan 31;15(1):e0228108. doi: 10.1371/journal.pone.0228108 (PMC6994199; doi:10.1371/journal.pone.0228108)
Supplement: S1 Table — For each single species cultures of 100 mL keratin liquid keratin was inoculated with 1 mL of an OD600nm = 0.7 adjusted culture. (a) CFU per mL at OD 0.7 for each single species. (b) CFU * mg-1 keratin with inoculation of 1 mL of a given single species culture at OD600nm = 0.7. (DOCX) [file pone.0228108.s001.docx]

| **Species** | **CFU/ mL ^(a)^** | **CFU per Keratin ^(b)^** |
| --- | --- | --- |
| *S. rhizophila* | 654630348 | 65463035 |
| *X. retroflexus* | 805162687 | 80516269 |
| *M. oxydans* | 3820554229 | 382055423 |
| *P. amylolyticus* | 23989987 | 2398999 |

S1 Table. Cell inoculation in keratin liquid media for keratin degradation. For each single species cultures of 100 mL keratin liquid keratin was inoculated with 1 mL of an OD_600nm_ = 0.7 adjusted culture. (a) CFU per mL at OD 0.7 for each single species. (b) CFU * mg^-1^ keratin with inoculation of 1 mL of a given single species culture at OD_600nm_ = 0.7.
