# Supplementary material for: Community-intrinsic properties enhance keratin degradation from bacterial consortia
Source: PLoS One. 2020 Jan 31;15(1):e0228108. doi: 10.1371/journal.pone.0228108 (PMC6994199; doi:10.1371/journal.pone.0228108)
Supplement: S3 Table — Protease.Act refers to measured protease activity, Keratinase.Act refers to measured keratinase activity, Protein.Conc refers to measured protein concentration in the culture supernatant, Total_CFU refers to the summed CFU counts for all species in the culture, KeratinLoss refers to amount of keratin removed in the culture during cultivation, and Biofilm refers to counts of 16S rDNA gene copies. (DOCX) [file pone.0228108.s003.docx]

| **Pairwise comparisons** | **Cor. Coef. (*r_s_*)** | **p-value** | **p_adj_** |
| --- | --- | --- | --- |
| Protease.Act - Keratinase.Act | 0.6 | 0.2847570 | 0.4745950 |
| Protease.Act - Protein.conc | 0.7 | 0.1881204 | 0.4031152 |
| Keratinase.Act - Protein.conc | 0.9 | 0.0373861 | 0.1401978 |
| Protease.Act - KeratinLoss | 0.4 | 0.5046316 | 0.6881340 |
| Keratinase.Act - KeratinLoss | 0.8 | 0.1040880 | 0.2602201 |
| Protein.conc - KeratinLoss | 0.9 | 0.0373861 | 0.1401978 |
| Protease.Act - Total_CFU | -0.2 | 0.7470601 | 0.8619924 |
| Keratinase.Act - Total_CFU | -0.6 | 0.2847570 | 0.4745950 |
| Protein.conc - Total_CFU | -0.2 | 0.7470601 | 0.8619924 |
| KeratinLoss - Total_CFU | -0.1 | 0.8728886 | 0.8728886 |
| Protease.Act - Biofilm | 0.4 | 0.5046316 | 0.6881340 |
| Keratinase.Act - Biofilm | 0.8 | 0.1040880 | 0.2602201 |
| Protein.conc - Biofilm | 0.9 | 0.0373861 | 0.1401978 |
| KeratinLoss - Biofilm | 1.0 | 0.01667 | 0.1401978 |
| Total_CFU - Biofilm | -0.1 | 0.8728886 | 0.8728886 |

S3 Table. Spearman’s ranked correlations coefficients and associated p-values and FDR adjusted p-values (p_adj_) for all variables include in Figure S15. Protease.Act refers to measured protease activity, Keratinase.Act refers to measured keratinase activity, Protein.Conc refers to measured protein concentration in the culture supernatant, Total_CFU refers to the summed CFU counts for all species in the culture, KeratinLoss refers to amount of keratin removed in the culture during cultivation, and Biofilm refers to counts of 16S rDNA gene copies.
