# Supplementary material for: Community-intrinsic properties enhance keratin degradation from bacterial consortia
Source: PLoS One. 2020 Jan 31;15(1):e0228108. doi: 10.1371/journal.pone.0228108 (PMC6994199; doi:10.1371/journal.pone.0228108)
Supplement: S4 Table — S. rhizophila, X. retroflexus, M. oxydans and P. amylolyticus are represented by the letters S, X, M and P, respectively. Co-cultures are represented by letters signifying it single species constituents, e.g. XS represents the co-culture of X. retroflexus and S. rhizophila. (DOCX) [file pone.0228108.s004.docx]

|  | Azo-casein | | |  | Azo-keratin | | |
| --- | --- | --- | --- | --- | --- | --- | --- |
| *Culture* | *Mean Slope* | *p_norm_* | *p_adj_* |  | *Mean Slope* | *p_norm_* | *p_adj_* |
| X | 5.8712 | NA | NA |  | 4.5834 | NA | NA |
| XS | 4.8847 | 0.1759 | 0.5387 |  | 6.0856 | 0.2975 | 0.7564 |
| XM | 6.7197 | 0.2437 | 0.6728 |  | 7.3489 | 0.0571 | 0.2095 |
| XP | 6.4435 | 0.4306 | 0.8949 |  | 7.274 | 0.064 | 0.2323 |
| 4-species | 7.8718 | 0.007 | 0.0276 |  | 8.0295 | 0.0184 | 0.0718 |

S4 Table. Mean slope coefficients and p-values for the liner model on azo-casein and azo-keratin degradation. *S. rhizophila, X. retroflexus, M. oxydans* and *P. amylolyticus* are represented by the letters S, X, M and P, respectively. Co-cultures are represented by letters signifying it single species constituents, e.g. XS represents the co-culture of *X. retroflexus* and *S. rhizophila*.
