# Supplementary material for: Community-intrinsic properties enhance keratin degradation from bacterial consortia
Source: PLoS One. 2020 Jan 31;15(1):e0228108. doi: 10.1371/journal.pone.0228108 (PMC6994199; doi:10.1371/journal.pone.0228108)
Supplement: S1 Fig — Over night cultures in TSB were diluted and plate spread on TSA for a set of fixed OD values. Each plating at a given OD was performed in duplicates. Solid lines corresponds to a linear regression across the data points, with the light grey area showing the rolling average of the 95% confidence interval across the data points. Single letters corresponds to the individual single species accordingly; X is Xanthomonas retroflexus, S is Stenotrophomonas rhizophila, M is Microbacterium oxydans and P is Paenibacillus amylolyticus. (DOCX) [file pone.0228108.s005.docx]

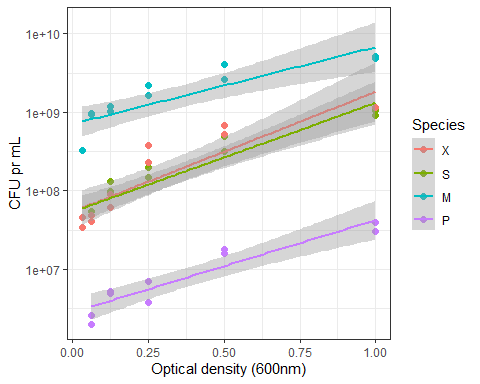


S1 Fig. CFU * mL^-1^ for each single species culture. Over night cultures in TSB were diluted and plate spread on TSA for a set of fixed OD values. Each plating at a given OD was performed in duplicates. Solid lines corresponds to a linear regression across the data points, with the light grey area showing the rolling average of the 95% confidence interval across the data points. Single letters corresponds to the individual single species accordingly; X is *Xanthomonas retroflexus*, S is *Stenotrophomonas rhizophila*, M is *Microbacterium oxydans* and P is *Paenibacillus amylolyticus*.
