# Supplementary material for: Community-intrinsic properties enhance keratin degradation from bacterial consortia
Source: PLoS One. 2020 Jan 31;15(1):e0228108. doi: 10.1371/journal.pone.0228108 (PMC6994199; doi:10.1371/journal.pone.0228108)
Supplement: S2 Fig — Identified proteins were filtered for the presence of signal peptides by SignalP, only including proteins which contained signal peptides. Principal component analysis was performed on Log2 transformed protein intensities using zero centering and unit variance scaling for the PCA analysis with the prcomb R-package. Biological replicates X and Y were clearly differentiating from the other biological replicates, which would hamper proper protein quantification. These two replicates were therefore excluded prior to further analysis. (DOCX) [file pone.0228108.s006.docx]

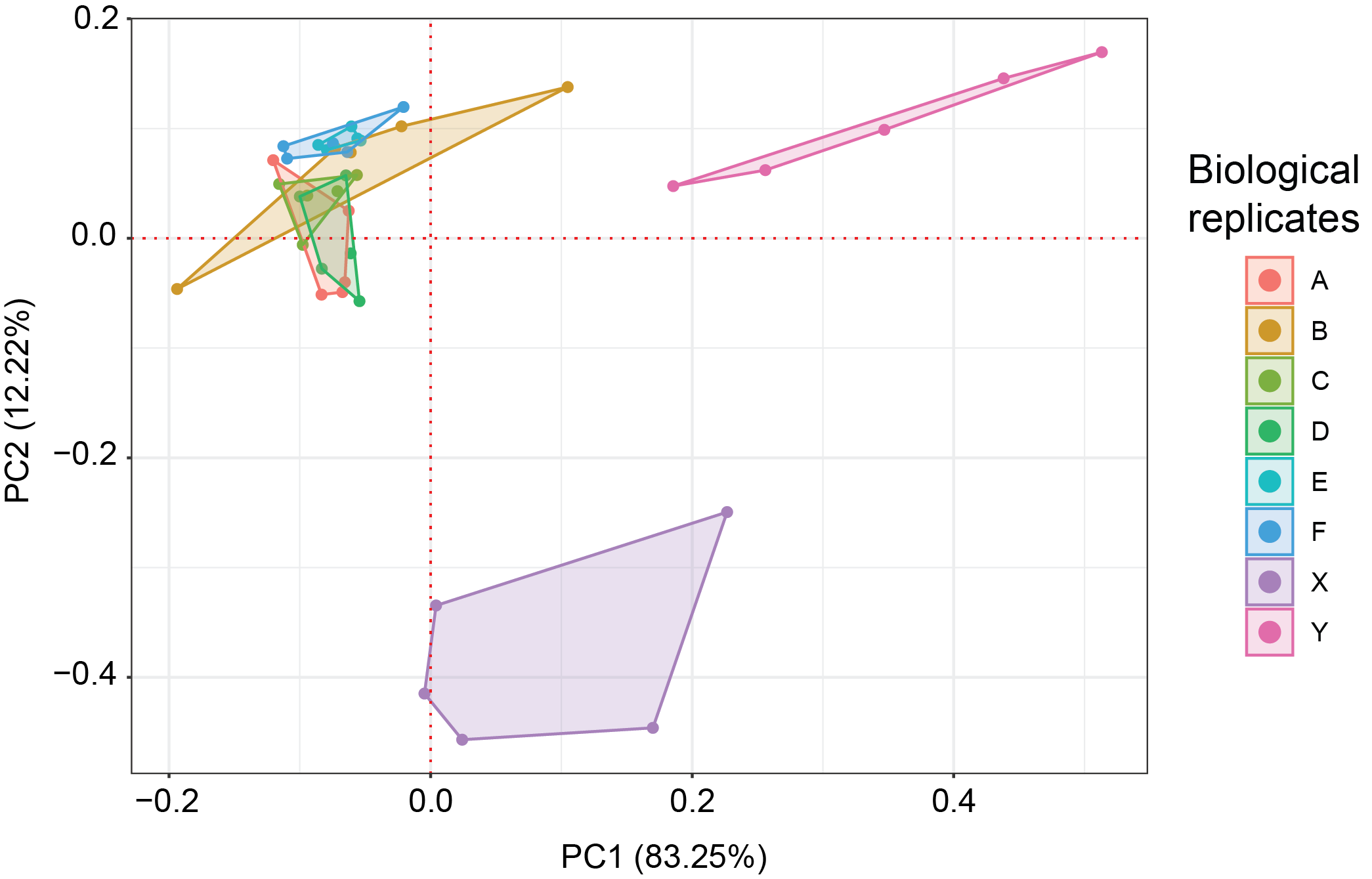


S2 Fig. Principal component analysis of biological replicates included in the protein identification from secretome profiling. Identified proteins were filtered for the presence of signal peptides by SignalP, only including proteins which contained signal peptides. Principal component analysis was performed on Log2 transformed protein intensities using zero centering and unit variance scaling for the PCA analysis with the prcomb R-package. Biological replicates X and Y were clearly differentiating from the other biological replicates, which would hamper proper protein quantification. These two replicates were therefore excluded prior to further analysis.
