# Supplementary material for: Community-intrinsic properties enhance keratin degradation from bacterial consortia
Source: PLoS One. 2020 Jan 31;15(1):e0228108. doi: 10.1371/journal.pone.0228108 (PMC6994199; doi:10.1371/journal.pone.0228108)
Supplement: S5 Fig — Numbers represents Pearson’s correlation between individual samples. The biological replicates X and Y had very low correlation scores with the other replicates, which could influence protein quantification. Similarly, the biological replicate B also represented very low correlation scores, indicating problems with either sample prep or mass spectrometry analysis. (DOCX) [file pone.0228108.s009.docx]

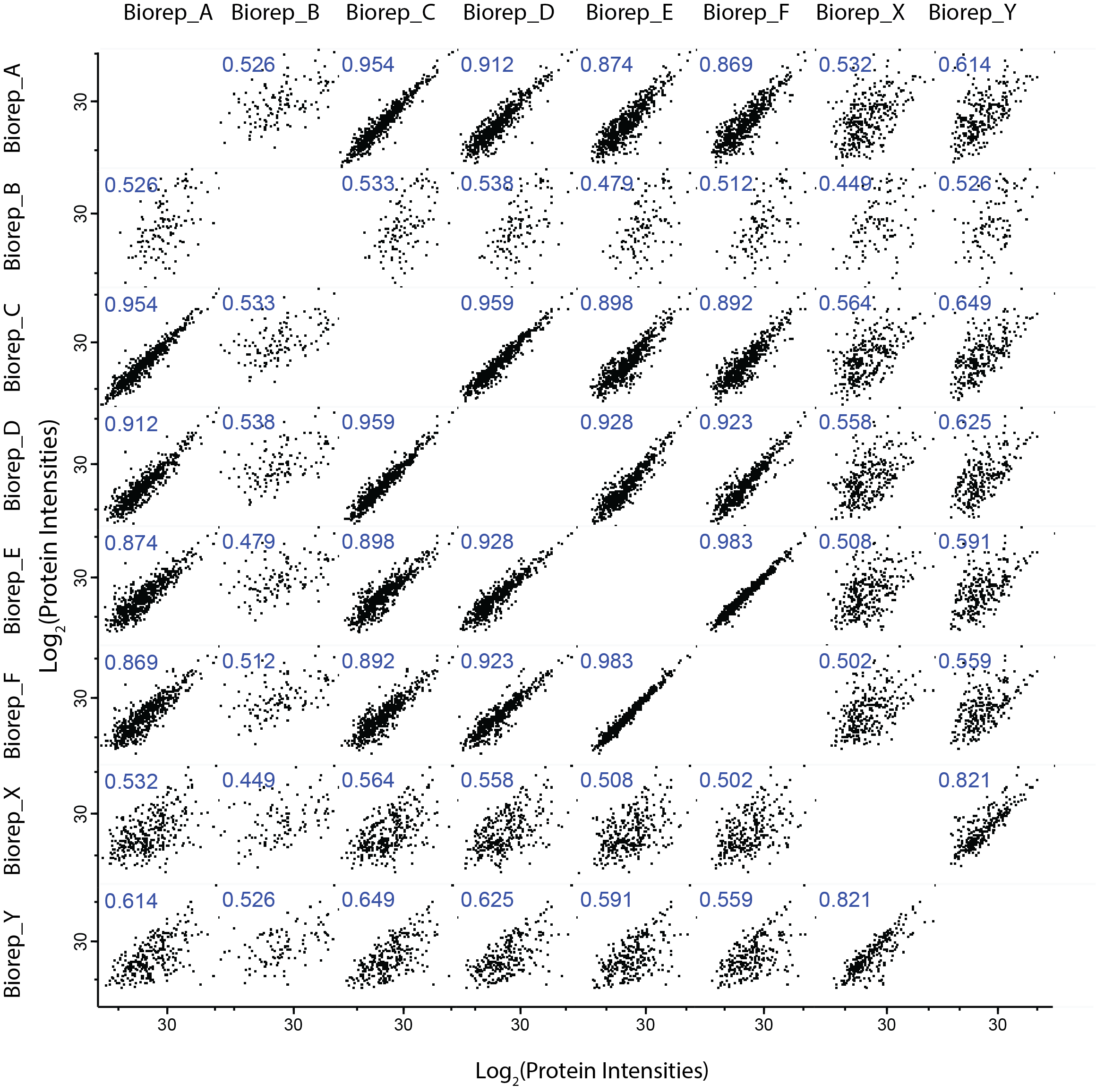


S5 Fig. Multi-scatter plot of biological replicates from the secretome profiling of *X. retroflexus – M. oxydans* cultures. Numbers represents Pearson’s correlation between individual samples. The biological replicates X and Y had very low correlation scores with the other replicates, which could influence protein quantification. Similarly, the biological replicate B also represented very low correlation scores, indicating problems with either sample prep or mass spectrometry analysis.
