# Supplementary material for: Community-intrinsic properties enhance keratin degradation from bacterial consortia
Source: PLoS One. 2020 Jan 31;15(1):e0228108. doi: 10.1371/journal.pone.0228108 (PMC6994199; doi:10.1371/journal.pone.0228108)
Supplement: S6 Fig — Numbers represents Pearson’s correlation between individual samples. The biological replicates X and Y had very low correlation scores with the other replicates, which could influence protein quantification. (DOCX) [file pone.0228108.s010.docx]

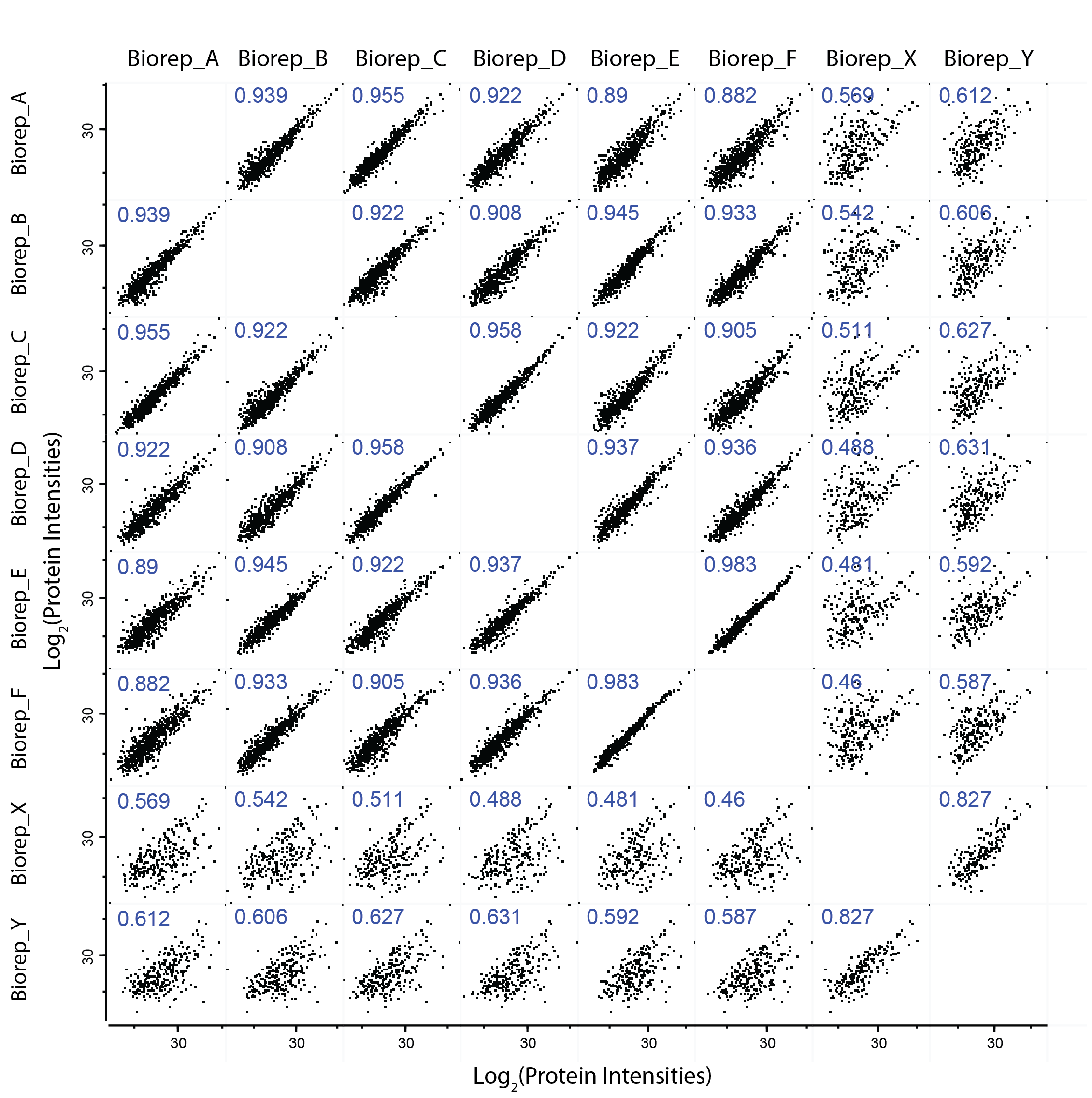


S6 Fig. Multi-scatter plot of biological replicates from the secretome profiling of *X. retroflexus – P. amylolyticus* cultures. Numbers represents Pearson’s correlation between individual samples. The biological replicates X and Y had very low correlation scores with the other replicates, which could influence protein quantification.
