# Supplementary material for: Community-intrinsic properties enhance keratin degradation from bacterial consortia
Source: PLoS One. 2020 Jan 31;15(1):e0228108. doi: 10.1371/journal.pone.0228108 (PMC6994199; doi:10.1371/journal.pone.0228108)
Supplement: S8 Fig — a) CFU counts of mono- and co-cultures. S. rhizophila, X. retroflexus, M. oxydans and P. amylolyticus are represented by the S, X, M and P, respectively. Co-cultures are represented by letter combinations of its single species constituents, e.g. XS represents the co-culture of X. retroflexus and S. rhizophila. Bars of mono-cultures represent the mean of three biological replicates with error bars showing standard deviation of the replicates. Stacked bars of co-cultures are the summed average of each species from three biological replicates with error bars displaying the standard deviation of the summed mean of all species in the co-culture. Statistical difference by a linear regression with post-hoc Tukey’s HSD pairwise hypothesis testing and single-step p-value correction (Lin.1). The red dotted line signifies separates mono and co-cultures. Mono-cultures were statistically compared to each other. Co-cultures were only statistically compared to X. retroflexus mono-culture. Statistical difference was found between some mono-cultures, as signified by dissimilar lettering (ascending order, padj < 0.05). No statistical difference was found between the summed averages of co-cultures and the average of the X. retroflexus mono-culture. b) CFU counts of P. amylolyticus as mono and co-cultures. Statistical difference was found between cultures, as signified by dissimilar lettering (padj < 0.05, Lin.1). (DOCX) [file pone.0228108.s012.docx]

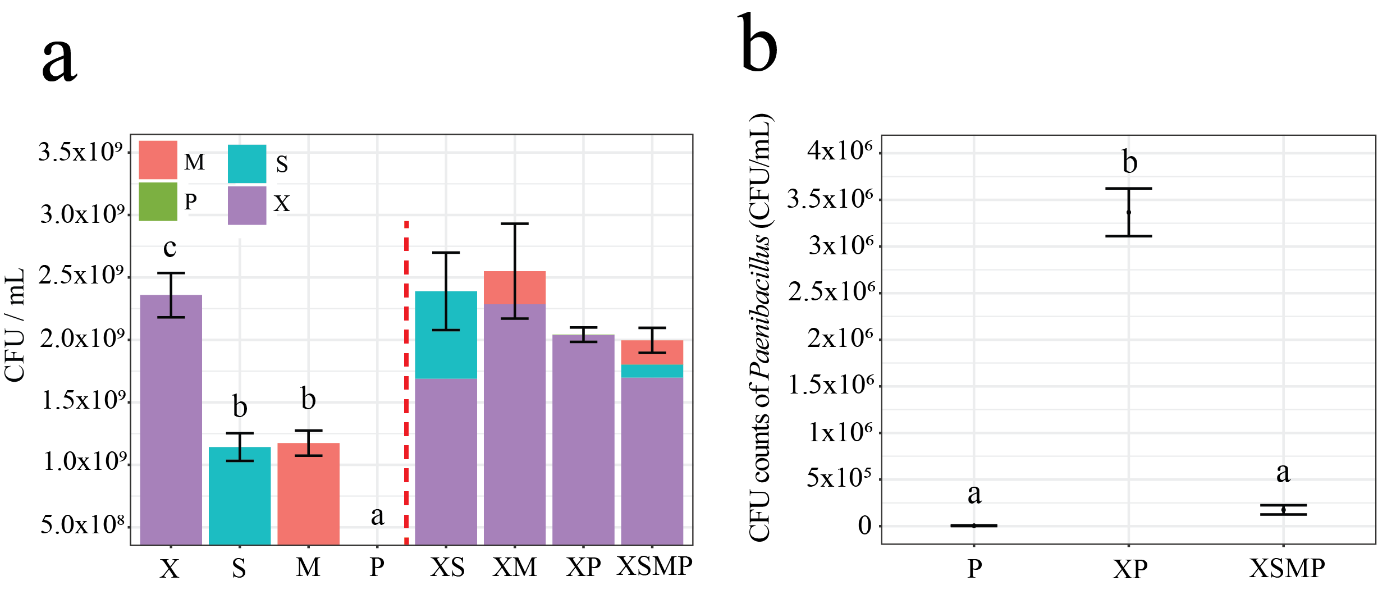


S8 Fig. Counts of colony forming units across culture types. a) CFU counts of mono- and co-cultures. *S. rhizophila*, *X. retroflexus, M. oxydans* and *P. amylolyticus* are represented by the S, X, M and P, respectively. Co-cultures are represented by letter combinations of its single species constituents, e.g. XS represents the co-culture of *X. retroflexus* and *S. rhizophila*. Bars of mono-cultures represent the mean of three biological replicates with error bars showing standard deviation of the replicates. Stacked bars of co-cultures are the summed average of each species from three biological replicates with error bars displaying the standard deviation of the summed mean of all species in the co-culture. Statistical difference by a linear regression with post-hoc Tukey’s HSD pairwise hypothesis testing and single-step p-value correction (Lin.1). The red dotted line signifies separates mono and co-cultures. Mono-cultures were statistically compared to each other. Co-cultures were only statistically compared to *X. retroflexus* mono-culture. Statistical difference was found between some mono-cultures, as signified by dissimilar lettering (ascending order, p_adj_ < 0.05). No statistical difference was found between the summed averages of co-cultures and the average of the *X. retroflexus* mono-culture.
b) CFU counts of *P. amylolyticus* as mono and co-cultures. Statistical difference was found between cultures, as signified by dissimilar lettering (p_adj_ < 0.05, Lin.1).
