# Supplementary material for: Community-intrinsic properties enhance keratin degradation from bacterial consortia
Source: PLoS One. 2020 Jan 31;15(1):e0228108. doi: 10.1371/journal.pone.0228108 (PMC6994199; doi:10.1371/journal.pone.0228108)
Supplement: S9 Fig — S. rhizophila, X. retroflexus, M. oxydans are represented by the S, X, and M respectively. Keratin degradation is calculated as pico-gram keratin degraded per CFU from the cultures. Point represents the mean of three biological replicates with error bars displaying standard deviation. Statistical significance was inferred by linear regression with post-hoc Tukey’s HSD pairwise hypothesis testing and single-step multiple step correction, as signified by dissimilar lettering (padj < 0.05, Lin.1). (DOCX) [file pone.0228108.s013.docx]

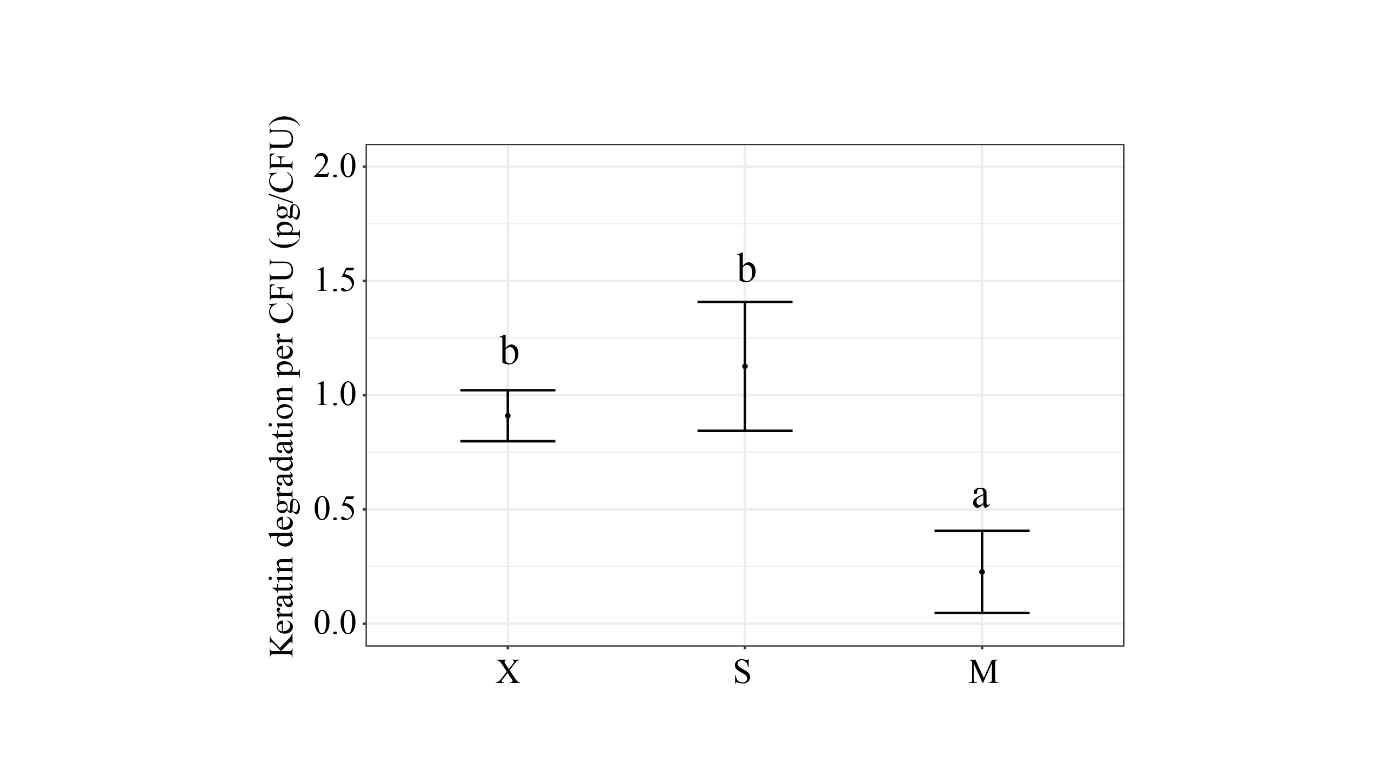


S9 Fig. Keratin degradation per CFU for *X. retroflexus*, *S. rhizophila* and *M. oxydans* as mono-cultures. *S. rhizophila, X. retroflexus, M. oxydans* are represented by the S, X, and M respectively. Keratin degradation is calculated as pico-gram keratin degraded per CFU from the cultures. Point represents the mean of three biological replicates with error bars displaying standard deviation. Statistical significance was inferred by linear regression with post-hoc Tukey’s HSD pairwise hypothesis testing and single-step multiple step correction, as signified by dissimilar lettering (p_adj_ < 0.05, Lin.1).
