# Supplementary material for: Community-intrinsic properties enhance keratin degradation from bacterial consortia
Source: PLoS One. 2020 Jan 31;15(1):e0228108. doi: 10.1371/journal.pone.0228108 (PMC6994199; doi:10.1371/journal.pone.0228108)
Supplement: S10 Fig — S. rhizophila, X. retroflexus, M. oxydans and P. amylolyticus are represented by the S, X, M and P, respectively. Co-cultures are represented by letter combinations of its single species constituents, e.g XS represents the co-culture of X. retroflexus and S. rhizophila. a) Keratin degradation by X. retroflexus mono and co-cultures. Mean of keratin degradation from three biological replicates, with error bars showing standard deviation. Statistical difference was inferred by a pair-wise comparison of co-culture to mono-culture by a linear regression p-value corrected by single-step method. Both nominal and adjusted p-values are displayed for tests having a nominal significant p-value. Means of co-cultures were as follows; X. retroflexus-S. rhizophila (2.6 ±0.42 mg/mL, std.dev), X. retroflexus-M. oxydans (2.8 ±0.38 mg/mL, std.dev), X. retroflexus-P. amylolyticus (2.9 ±0.21 mg/mL, std.dev) and four-species community (XSMP) (2.7 ±0.12 mg/mL, std.dev). Both nominal and adjusted p-values are displayed for tests having a nominal significant p-value. b) Keratin degradation per CFU by X. retroflexus mono and co-cultures. Keratin degraded per CFU was calculated as the total amount of measured keratin degraded in the culture, divided by the total count of CFU from the culture. Mean of keratin degradation from three biological replicates, with error bars showing standard deviation. Statistical difference was inferred by a linear regression model (Lin.1). c) Fold-change in keratin degradation per CFU by co-cultures of X. retroflexus, related to the X. retroflexus mono-culture (indicated by dotted red line). Statistical difference was inferred by Lin.3. (DOCX) [file pone.0228108.s014.docx]

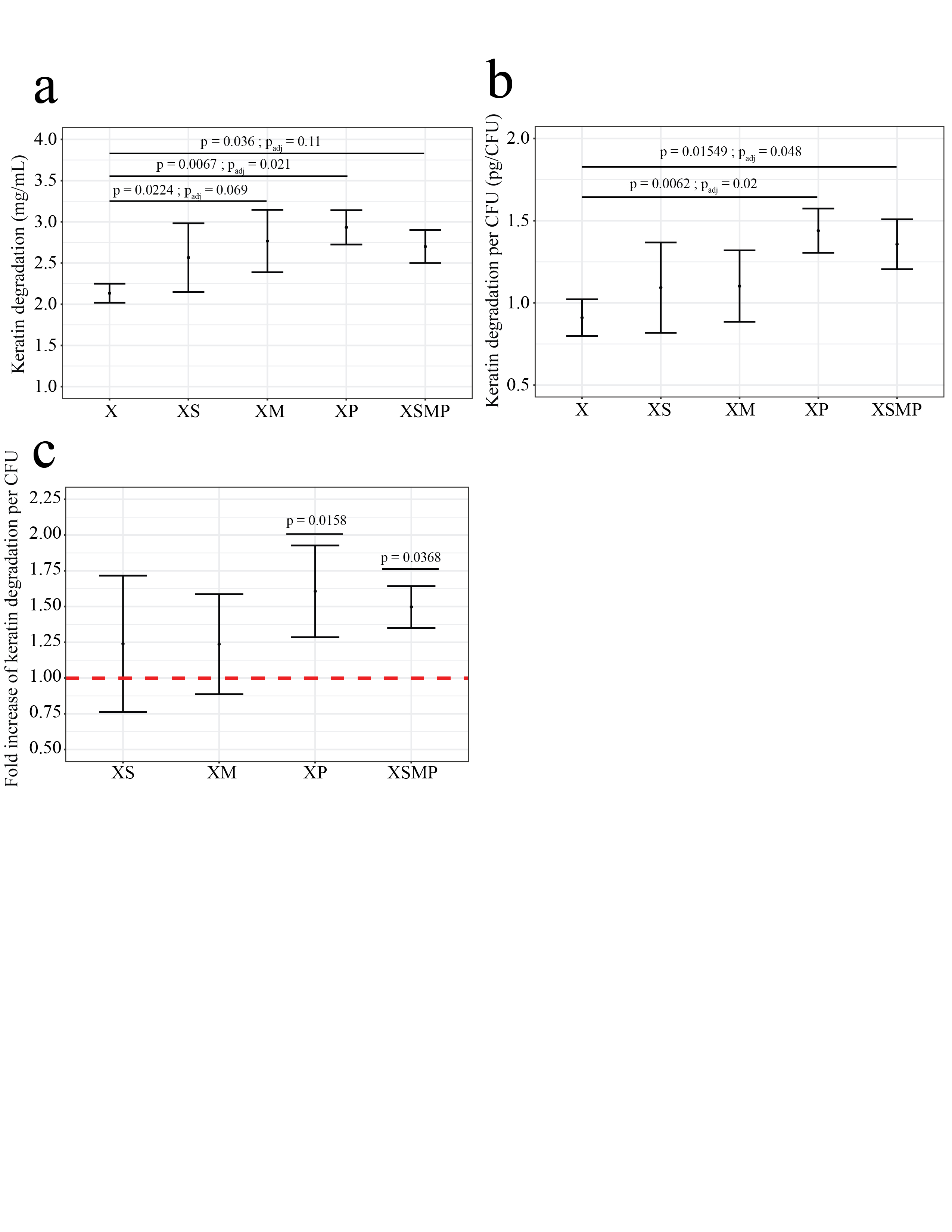


S10 Fig. Keratin degradation with and without CFU correction for Co-cultures of *X. retroflexus*. *S. rhizophila, X. retroflexus, M. oxydans and P. amylolyticus* are represented by the S, X, M and P, respectively. Co-cultures are represented by letter combinations of its single species constituents, e.g XS represents the co-culture of *X. retroflexus* and *S. rhizophila*.
a) Keratin degradation by *X. retroflexus* mono and co-cultures. Mean of keratin degradation from three biological replicates, with error bars showing standard deviation. Statistical difference was inferred by a pair-wise comparison of co-culture to mono-culture by a linear regression p-value corrected by single-step method. Both nominal and adjusted p-values are displayed for tests having a nominal significant p-value. Means of co-cultures were as follows; *X. retroflexus-S. rhizophila* (2.6 ±0.42 mg/mL, std.dev), *X. retroflexus-M. oxydans* (2.8 ±0.38 mg/mL, std.dev), *X. retroflexus-P. amylolyticus* (2.9 ±0.21 mg/mL, std.dev) and four-species community (XSMP) (2.7 ±0.12 mg/mL, std.dev). Both nominal and adjusted p-values are displayed for tests having a nominal significant p-value.
b) Keratin degradation per CFU by *X. retroflexus* mono and co-cultures. Keratin degraded per CFU was calculated as the total amount of measured keratin degraded in the culture, divided by the total count of CFU from the culture. Mean of keratin degradation from three biological replicates, with error bars showing standard deviation. Statistical difference was inferred by a linear regression model (Lin.1).
c) Fold-change in keratin degradation per CFU by co-cultures of *X. retroflexus*, related to the *X. retroflexus* mono-culture (indicated by dotted red line). Statistical difference was inferred by Lin.3.
