# Supplementary material for: Community-intrinsic properties enhance keratin degradation from bacterial consortia
Source: PLoS One. 2020 Jan 31;15(1):e0228108. doi: 10.1371/journal.pone.0228108 (PMC6994199; doi:10.1371/journal.pone.0228108)
Supplement: S11 Fig — S. rhizophila, X. retroflexus, M. oxydans and P. amylolyticus are represented by the letters S, X, M and P, respectively. Co-cultures are represented by letters signifying it single species constituents, e.g. XS represents the co-culture of X. retroflexus and S. rhizophila. Error bars represent standard deviation of three biological replicates. Significant difference was inferred by independent two-sample t-test. ‘Measured’ refers to the experimentally measured keratin degradation. a) Comparison of measured degradation, for mono and co-cultures of X. retroflexus, to the theoretical amount of potential keratin degradation. The theoretical value refers to the sum of keratin degraded by each of the individual single-species cultures constituting the co-culture. b) Keratin degradation per CFU by mono- and co- cultures of X. retroflexus. Expected value refers to the theoretical amount of keratin degraded by co-cultures calculated as follows; the theoretical amount of keratin to be degraded by a given co-culture was estimated as the sum of keratin, which could be degraded by the amount of CFU from each species observed in the given culture. The amount degraded by each species was inferred from the potential of the respective mono-species cultures. The summed keratin degradation was then normalised against the total CFU from the respective co-cultures. (DOCX) [file pone.0228108.s015.docx]

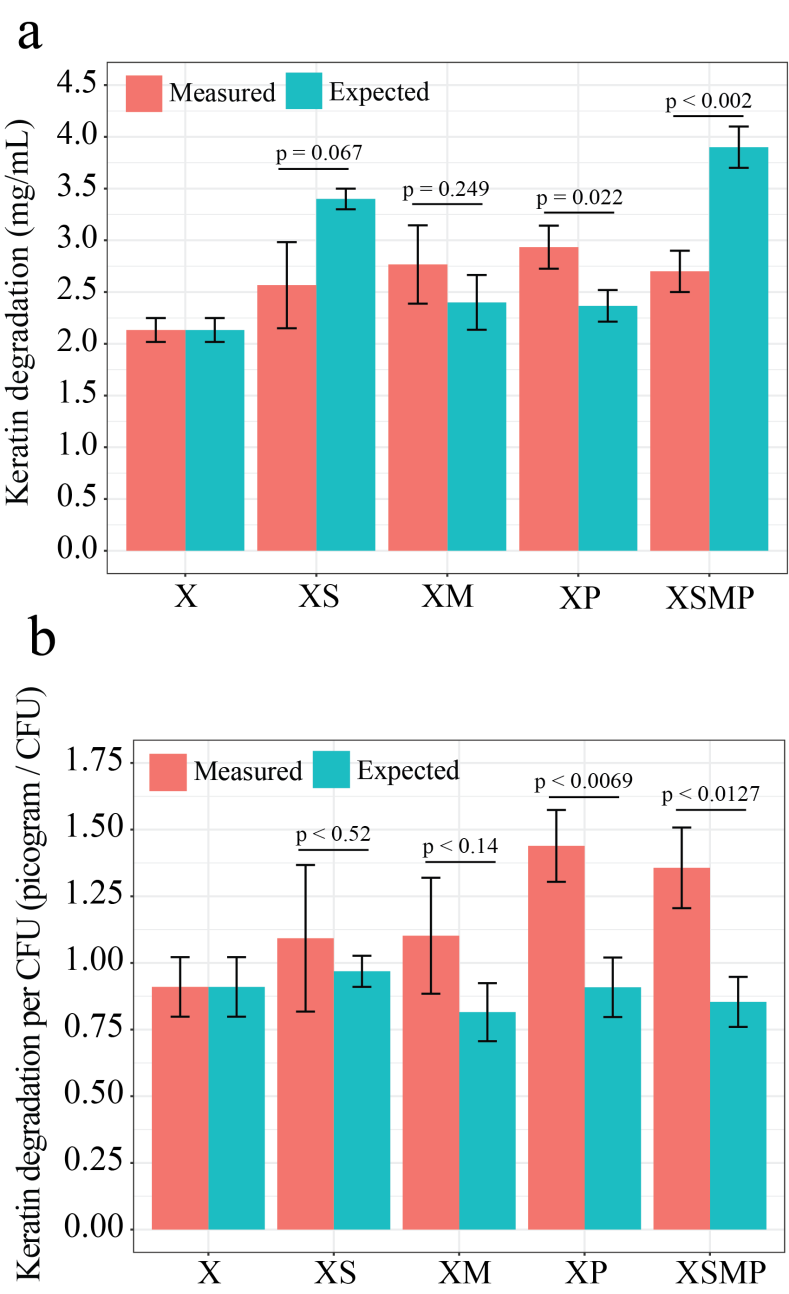


S11 Fig. Measured and expected theoretical keratin degradation from *X. retroflexus* mono and co-cultures, with and without correction for species composition and CFU counts. *S. rhizophila*, *X. retroflexus*, *M. oxydans* and *P. amylolyticus* are represented by the letters S, X, M and P, respectively. Co-cultures are represented by letters signifying it single species constituents, e.g. XS represents the co-culture of *X. retroflexus* and *S. rhizophila*. Error bars represent standard deviation of three biological replicates. Significant difference was inferred by independent two-sample t-test. ‘Measured’ refers to the experimentally measured keratin degradation.
a) Comparison of measured degradation, for mono and co-cultures of *X. retroflexus,* to the theoretical amount of potential keratin degradation. The theoretical value refers to the sum of keratin degraded by each of the individual single-species cultures constituting the co-culture.
b) Keratin degradation per CFU by mono- and co- cultures of *X. retroflexus*. Expected value refers to the theoretical amount of keratin degraded by co-cultures calculated as follows; the theoretical amount of keratin to be degraded by a given co-culture was estimated as the sum of keratin, which could be degraded by the amount of CFU from each species observed in the given culture. The amount degraded by each species was inferred from the potential of the respective mono-species cultures. The summed keratin degradation was then normalised against the total CFU from the respective co-cultures.
