# Supplementary material for: Community-intrinsic properties enhance keratin degradation from bacterial consortia
Source: PLoS One. 2020 Jan 31;15(1):e0228108. doi: 10.1371/journal.pone.0228108 (PMC6994199; doi:10.1371/journal.pone.0228108)
Supplement: S12 Fig — (DOCX) [file pone.0228108.s016.docx]

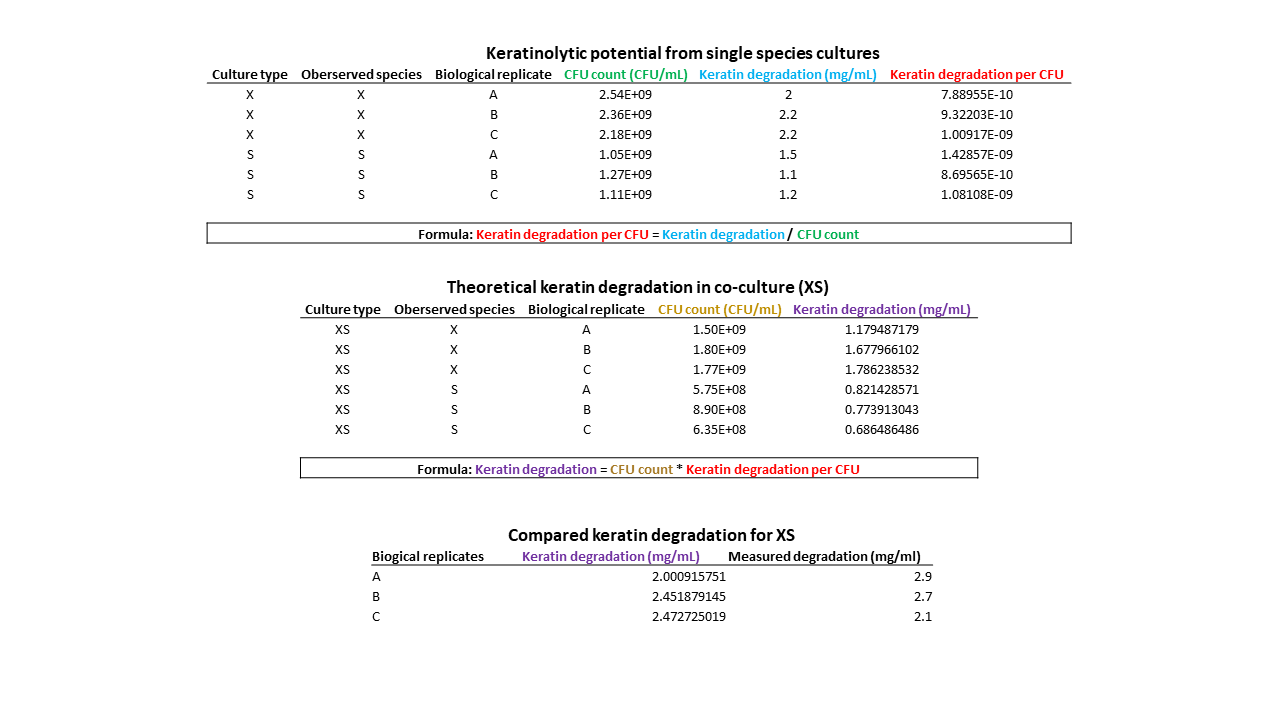


S12 Fig. Calculation of expected theoretical keratin degradation for the *X. retroflexus - S. rhizophila* co-culture from their individual single species degradation profiles.
