# Supplementary material for: Community-intrinsic properties enhance keratin degradation from bacterial consortia
Source: PLoS One. 2020 Jan 31;15(1):e0228108. doi: 10.1371/journal.pone.0228108 (PMC6994199; doi:10.1371/journal.pone.0228108)
Supplement: S13 Fig — Performed on keratin particles isolated from mono- and co-cultures at both 2 and 4 days of incubation. Copy numbers are believed to correspond to cells associated to the particles in a potential biofilm state. Mono- and co-cultures are represented by letters of their respective species; e.g. X. retroflexus (X), S. rhizophila (S), M. oxydans (M) and P. amylolyticus (P) for mono-cultures and e.g. X. retroflexus–M. oxydans (XM) for co-cultures. Statistical difference was inferred by Lin.1, as signified by dissimilar lettering (padj < 0.05). a) Counts from mono-cultures after 2 and 4 days of incubation. X. retroflexus produced significantly higher numbers than any of the other mono-cultures. b) Counts after 2 and 4 days of incubation from co-cultures. No significant difference was observed between counts from cultures after 2 and 4 days of incubation. After 4 days of incubation all co-cultures trended a higher level of counts than the X. retroflexus mono-culture. (DOCX) [file pone.0228108.s017.docx]

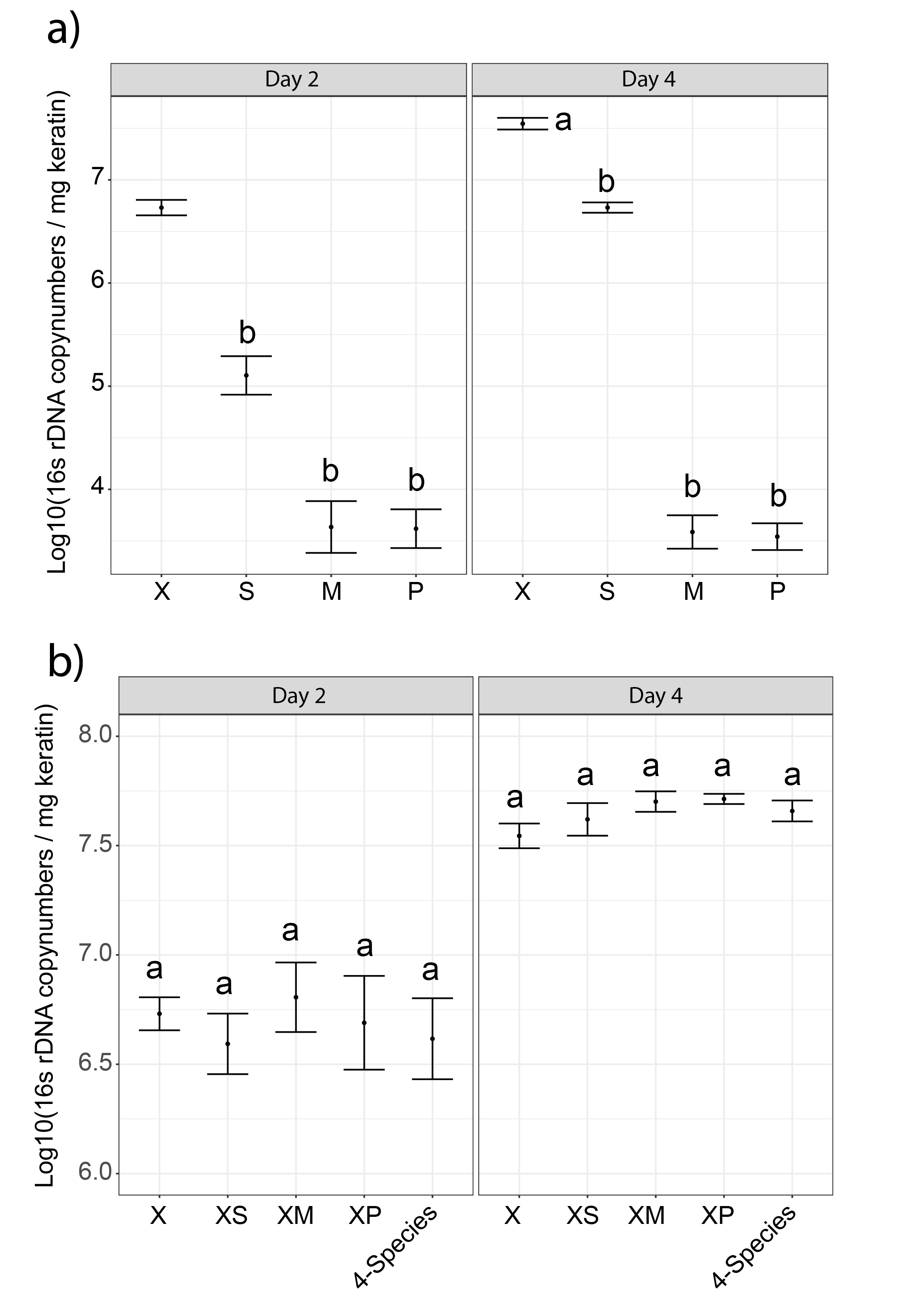


S13 Fig. Copy numbers from Q-PCR analysis, based on universal eubacterial 16s rDNA primers. Performed on keratin particles isolated from mono- and co-cultures at both 2 and 4 days of incubation. Copy numbers are believed to correspond to cells associated to the particles in a potential biofilm state. Mono- and co-cultures are represented by letters of their respective species; e.g. *X. retroflexus* (X), *S. rhizophila* (S), *M. oxydans* (M) *and P. amylolyticus* (P) for mono-cultures and e.g. *X. retroflexus – M. oxydans* (XM) for co-cultures. Statistical difference was inferred by Lin.1, as signified by dissimilar lettering (p_adj_ < 0.05).
a) Counts from mono-cultures after 2 and 4 days of incubation. *X. retroflexus* produced significantly higher numbers than any of the other mono-cultures.
b) Counts after 2 and 4 days of incubation from co-cultures. No significant difference was observed between counts from cultures after 2 and 4 days of incubation. After 4 days of incubation all co-cultures trended a higher level of counts than the *X. retroflexus* mono-culture.
