# Supplementary material for: Community-intrinsic properties enhance keratin degradation from bacterial consortia
Source: PLoS One. 2020 Jan 31;15(1):e0228108. doi: 10.1371/journal.pone.0228108 (PMC6994199; doi:10.1371/journal.pone.0228108)
Supplement: S15 Fig — Averaged values of all biological replicates across each culture type after 4 days of cultivation was used for the correlation analysis. Protease.Act refers to measured protease activity, Keratinase.Act refers to measured keratinase activity, Protein.Conc refers to measured protein concentration in the culture supernatant, Total_CFU refers to the summed CFU counts for all species in the culture, KeratinLoss refers to amount of keratin removed in the culture during cultivation, and Biofilm refers to counts of 16S rDNA gene copies. Color intensity and circle size corresponds to the size of the correlation coefficient ranging between -1 and 1. The larger and darker blue the circle is displayed the closer the correlation coefficient is t 1. The larger and darker red the circle is displayed, the closer the correlation coefficient is to -1. Stars within the circles correspond to level of significance for the FDR corrected p-values: no star refers to Padj > 0.05, * refers to 0.05 > Padj < 0.01, ** refers to 0.01 > Padj < 0.001 and *** refers to 0.001 > Padj. (DOCX) [file pone.0228108.s019.docx]

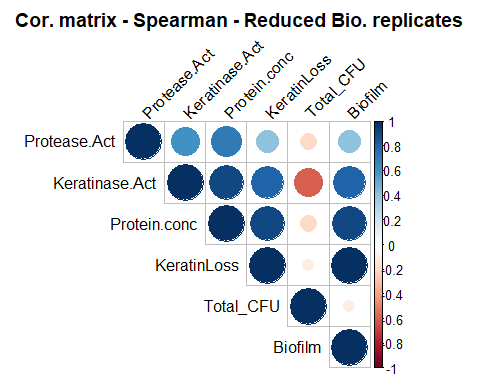


S15 Fig. Correlation matrix with Spearman’s ranked correlations between investigated variables. Averaged values of all biological replicates across each culture type after 4 days of cultivation was used for the correlation analysis. Protease.Act refers to measured protease activity, Keratinase.Act refers to measured keratinase activity, Protein.Conc refers to measured protein concentration in the culture supernatant, Total_CFU refers to the summed CFU counts for all species in the culture, KeratinLoss refers to amount of keratin removed in the culture during cultivation, and Biofilm refers to counts of 16S rDNA gene copies. Color intensity and circle size corresponds to the size of the correlation coefficient ranging between -1 and 1. The larger and darker blue the circle is displayed the closer the correlation coefficient is t 1. The larger and darker red the circle is displayed, the closer the correlation coefficient is to -1. Stars within the circles correspond to level of significance for the FDR corrected p-values: no star refers to P_adj_ > 0.05, * refers to 0.05 > P_adj_ < 0.01, ** refers to 0.01 > P_adj_ < 0.001 and *** refers to 0.001 > P_adj_.
