# Supplementary material for: Community-intrinsic properties enhance keratin degradation from bacterial consortia
Source: PLoS One. 2020 Jan 31;15(1):e0228108. doi: 10.1371/journal.pone.0228108 (PMC6994199; doi:10.1371/journal.pone.0228108)
Supplement: S16 Fig — Three biological replicates with lines representing linear regressions. a) Protease production by different combinations using azo-casein as substrate. One unit (U) protease activity was defined as the amount of protein that increased the absorbance by 0.01 under given conditions. b) Keratinase production by different combinations using azo-keratin as substrate. One unit keratinase activity was defined as the amount of protein that increased the absorbance by 0.01 under given conditions. c) Protein production from degraded keratin by different combinations. Bradford assay was used for protein quantification with BSA as standard. The higher initial protein concentration in M. oxydans and P. amylolyticus (also found in the blank media) reflects the presence of other proteins apart from keratin in the solution, which may be due to use of crude keratin. Since M. oxydans and P. amylolyticus do neither utilize nor degrade protein from the supernatant, they have the initial and almost same protein level during the entire incubation period. (DOCX) [file pone.0228108.s020.docx]

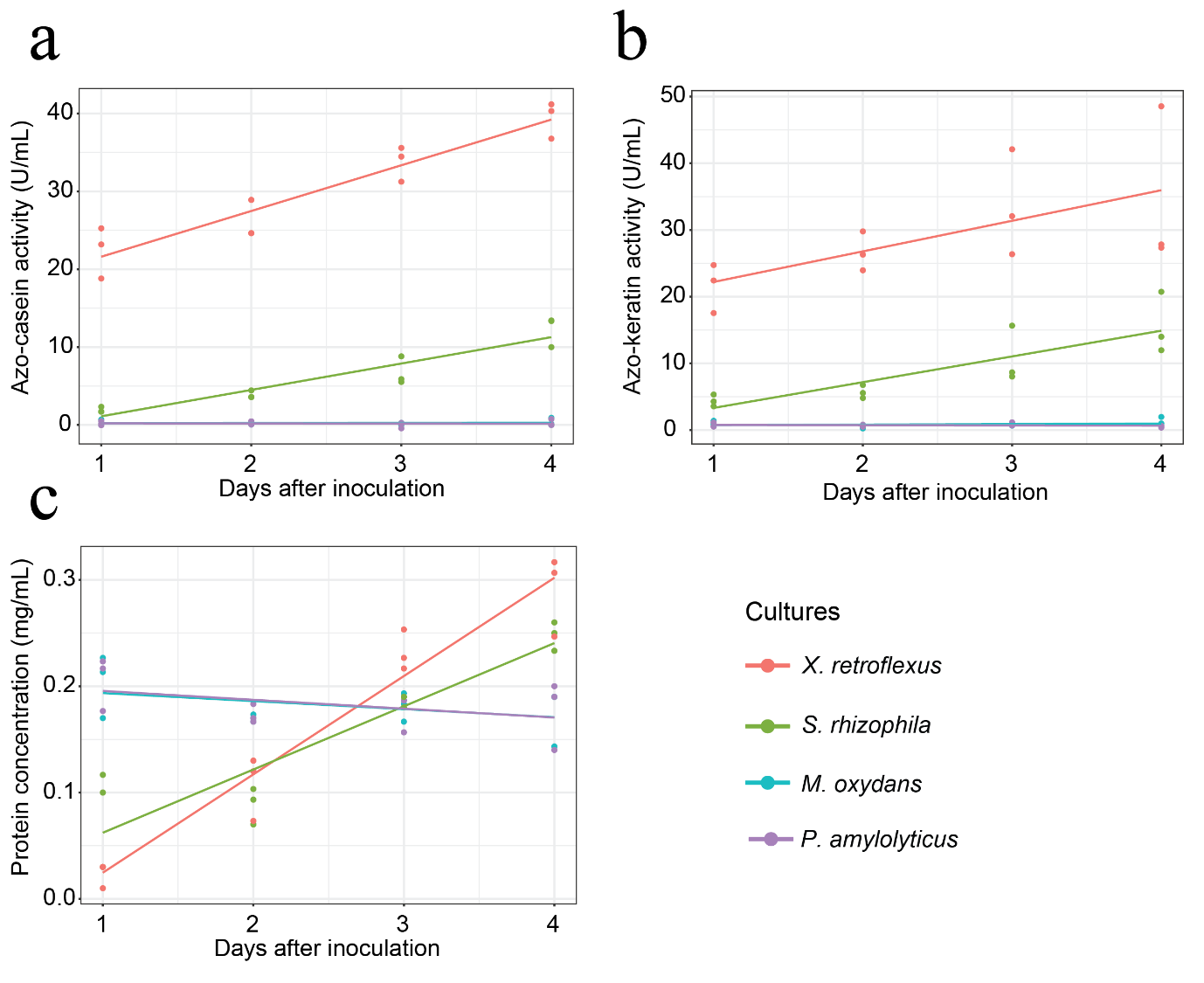


S16 Fig. Enzyme and protein production during growth by the mono-species cultures. Three biological replicates with lines representing linear regressions. a) Protease production by different combinations using azo-casein as substrate. One unit (U) protease activity was defined as the amount of protein that increased the absorbance by 0.01 under given conditions. b) Keratinase production by different combinations using azo-keratin as substrate. One unit keratinase activity was defined as the amount of protein that increased the absorbance by 0.01 under given conditions. c) Protein production from degraded keratin by different combinations. Bradford assay was used for protein quantification with BSA as standard. The higher initial protein concentration in *M. oxydans* and *P. amylolyticus* (also found in the blank media) reflects the presence of other proteins apart from keratin in the solution, which may be due to use of crude keratin. Since *M. oxydans* and *P. amylolyticus* do neither utilize nor degrade protein from the supernatant, they have the initial and almost same protein level during the entire incubation period.
