# Supplementary material for: Community-intrinsic properties enhance keratin degradation from bacterial consortia
Source: PLoS One. 2020 Jan 31;15(1):e0228108. doi: 10.1371/journal.pone.0228108 (PMC6994199; doi:10.1371/journal.pone.0228108)
Supplement: S17 Fig — Data was filtered to remove the two outlying biological replicates followed by a removal of one outlying technical replicate of the X. retroflexus–M. oxydans and X. retroflexus–S. rhizophila culture, respectively, where the sample analysis on the mass spectrometer had not yielded data of sufficient quality. Identified proteins were filtered for the presence of signal peptides by SignalP, only including proteins which contained signal peptides. Principal component analysis was performed on Log2 transformed protein intensities using zero centering and unit variance scaling for the PCA analysis with the prcomb R-package. Although some group overlap was observed, the co-cultures of X. retroflexus–M. oxydans and the four-species culture separated somewhat from the cultures of X. retroflexus and X. retroflexus–S. rhizophila on PCA2. (DOCX) [file pone.0228108.s021.docx]

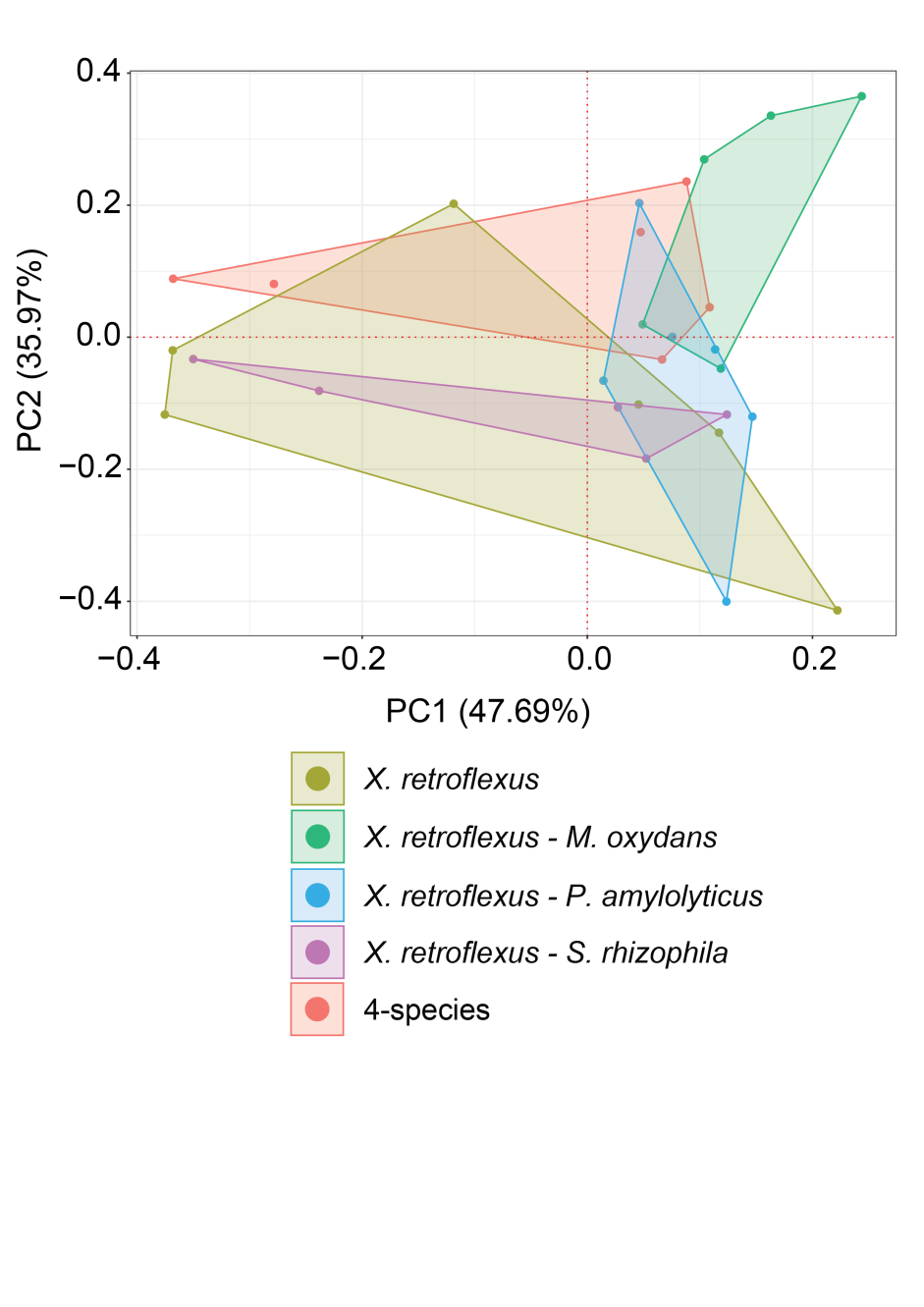
 S17 Fig. Principal component analysis of culture types from the secretome analysis of *X. retroflexus*. Data was filtered to remove the two outlying biological replicates followed by a removal of one outlying technical replicate of the *X. retroflexus – M. oxydans* and *X. retroflexus – S. rhizophila* culture, respectively, where the sample analysis on the mass spectrometer had not yielded data of sufficient quality. Identified proteins were filtered for the presence of signal peptides by SignalP, only including proteins which contained signal peptides. Principal component analysis was performed on Log2 transformed protein intensities using zero centering and unit variance scaling for the PCA analysis with the prcomb R-package. Although some group overlap was observed, the co-cultures of *X. retroflexus – M. oxydans* and the four-species culture separated somewhat from the cultures of *X. retroflexus* and *X. retroflexus – S. rhizophila* on PCA2.
