# Supplementary material for: Community-intrinsic properties enhance keratin degradation from bacterial consortia
Source: PLoS One. 2020 Jan 31;15(1):e0228108. doi: 10.1371/journal.pone.0228108 (PMC6994199; doi:10.1371/journal.pone.0228108)
Supplement: S18 Fig — Data was filtered to remove the two outlying biological replicates followed by a removal of one outlying technical replicate of the X. retroflexus–M. oxydans and X. retroflexus–S. rhizophila culture, respectively, where the sample analysis on the mass spectrometer had not yielded data of sufficient quality. Identified proteins were filtered for the presence of signal peptides by SignalP, only including proteins which contained signal peptides. Principal component analysis was performed on Log2 transformed protein intensities using zero centering and unit variance scaling for the PCA analysis with the prcomb R-package. The top ten protein variables having the largest effect on PCA1 and PCA2 in both positive and negative direction were extracted and mapped with MEROPS and RAST pathway function. Proteins without labels were hypothetical proteins from the RAST database without any known MEROPS function. PCA1 is most strongly influenced by the presence of a Chitinase (Fig|305959.5.peg.995). The effect of the Chitinase could mostly be explained by its variation in presence and absence between groups. PCA2 was strongly influenced by the variating abundance of three proteases. Two S08A serine proteases (fig|305959.5.peg.2700 and fig|305959.5.peg.3465) and a M72 metallo-endopeptidase (fig|305959.5.peg.2077) (DOCX) [file pone.0228108.s022.docx]

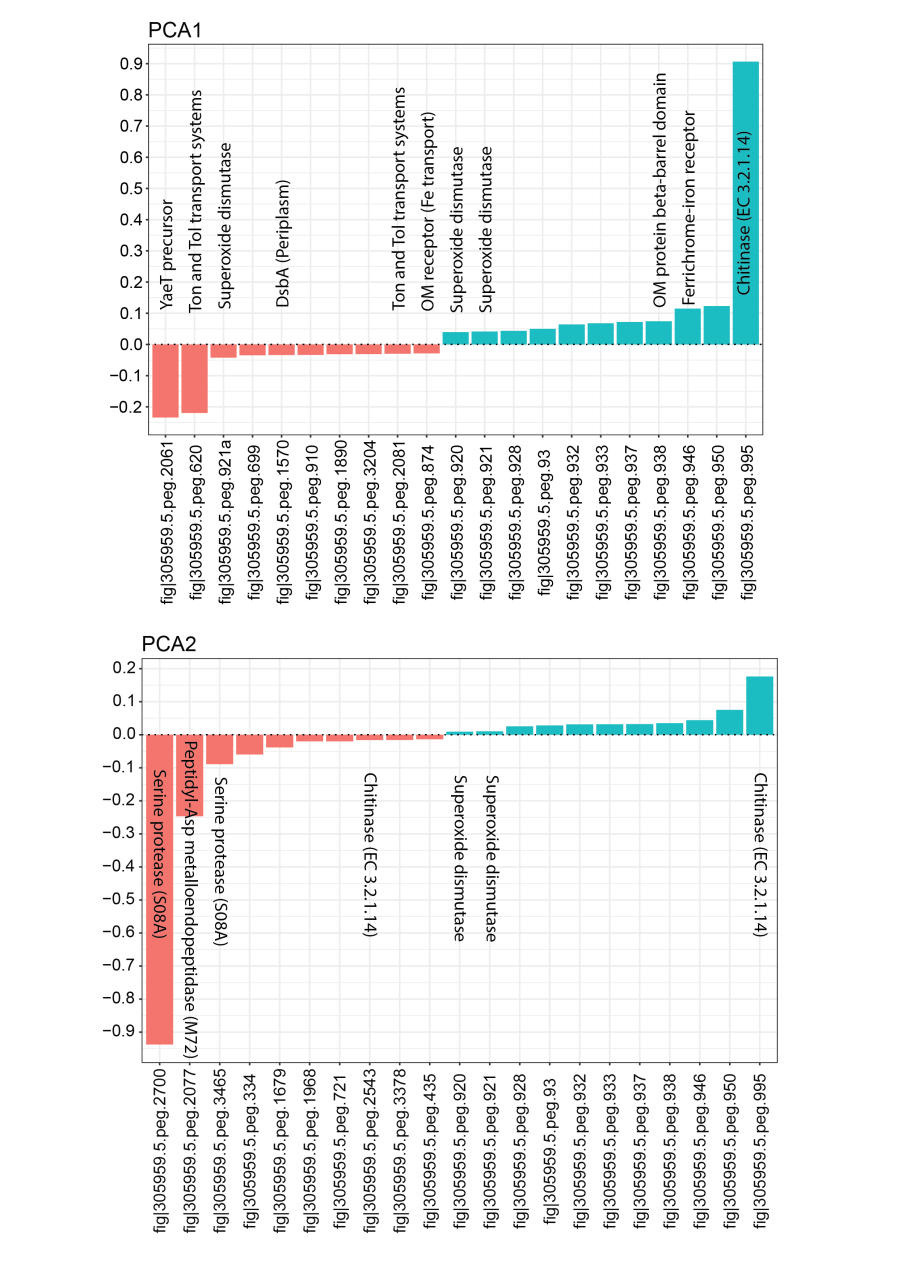
S18 Fig. Influence of top ten variables from the top two loadings from the principal component analysis. Data was filtered to remove the two outlying biological replicates followed by a removal of one outlying technical replicate of the *X. retroflexus – M. oxydans* and *X. retroflexus – S. rhizophila* culture, respectively, where the sample analysis on the mass spectrometer had not yielded data of sufficient quality. Identified proteins were filtered for the presence of signal peptides by SignalP, only including proteins which contained signal peptides. Principal component analysis was performed on Log2 transformed protein intensities using zero centering and unit variance scaling for the PCA analysis with the prcomb R-package. The top ten protein variables having the largest effect on PCA1 and PCA2 in both positive and negative direction were extracted and mapped with MEROPS and RAST pathway function. Proteins without labels were hypothetical proteins from the RAST database without any known MEROPS function. PCA1 is most strongly influenced by the presence of a Chitinase (Fig|305959.5.peg.995). The effect of the Chitinase could mostly be explained by its variation in presence and absence between groups (data not shown). PCA2 was strongly influenced by the variating abundance of three proteases. Two S08A serine proteases (fig|305959.5.peg.2700 and fig|305959.5.peg.3465) and a M72 metallo-endopeptidase (fig|305959.5.peg.2077)
