# Supplementary material for: Community-intrinsic properties enhance keratin degradation from bacterial consortia
Source: PLoS One. 2020 Jan 31;15(1):e0228108. doi: 10.1371/journal.pone.0228108 (PMC6994199; doi:10.1371/journal.pone.0228108)
Supplement: Attachment — Submitted filename: ResponseToReviewers.docx [file pone.0228108.s023.docx]

PONE-D-19-26241
Community-intrinsic properties enhance keratin degradation from bacterial consortia
PLOS ONE

Dear Dr. Sørensen,

Thank you for submitting your manuscript to PLOS ONE. After careful consideration, we feel that it has merit but does not fully meet PLOS ONE’s publication criteria as it currently stands. Therefore, we invite you to submit a revised version of the manuscript that addresses the points raised during the review process.

We would appreciate receiving your revised manuscript by Dec 15 2019 11:59PM. When you are ready to submit your revision, log on to <https://www.editorialmanager.com/pone/> and select the 'Submissions Needing Revision' folder to locate your manuscript file.

If you would like to make changes to your financial disclosure, please include your updated statement in your cover letter.

To enhance the reproducibility of your results, we recommend that if applicable you deposit your laboratory protocols in [protocols.io](http://protocols.io), where a protocol can be assigned its own identifier (DOI) such that it can be cited independently in the future. For instructions see: <http://journals.plos.org/plosone/s/submission-guidelines#loc-laboratory-protocols>

Please include the following items when submitting your revised manuscript:

- A rebuttal letter that responds to each point raised by the academic editor and reviewer(s). This letter should be uploaded as separate file and labeled 'Response to Reviewers'.
- A marked-up copy of your manuscript that highlights changes made to the original version. This file should be uploaded as separate file and labeled 'Revised Manuscript with Track Changes'.
- An unmarked version of your revised paper without tracked changes. This file should be uploaded as separate file and labeled 'Manuscript'.

Please note while forming your response, if your article is accepted, you may have the opportunity to make the peer review history publicly available. The record will include editor decision letters (with reviews) and your responses to reviewer comments. If eligible, we will contact you to opt in or out.

We look forward to receiving your revised manuscript.

Kind regards,

Arun K. Bhunia
Academic Editor
PLOS ONE

-----------------------------------------------------------------------------------------------------------------------

Dear editor and reviewers,

First, we would like to thank you for having reviewed our manuscript. The comments were very relevant and proved useful during the rewriting of our study. Please find the point-by-point response to the comments by reviewers and editor, with indications on how we included their remarks to the new version. To allow easy access to our changes, according to the comments by the reviewers, we have added new line references to our comments. Note that these line references correspond to the manuscript version with track changes.

Journal Requirements:

1. When submitting your revision, we need you to address these additional requirements. Please ensure that your manuscript meets PLOS ONE's style requirements, including those for file naming. The PLOS ONE style templates can be found at <http://www.journals.plos.org/plosone/s/file?id=wjVg/PLOSOne_formatting_sample_main_body.pdf> and <http://www.journals.plos.org/plosone/s/file?id=ba62/PLOSOne_formatting_sample_title_authors_affiliations.pdf>

Response: We have adjusted the manuscript accordingly.

2.  Please include captions for your Supporting Information files at the end of your manuscript, and update any in-text citations to match accordingly. Please see our Supporting Information guidelines for more information: <http://journals.plos.org/plosone/s/supporting-information>.
Response: All captions have been added to the manuscript.

3. We note that you have included the phrase “data not shown” in your manuscript. Unfortunately, this does not meet our data sharing requirements. PLOS does not permit references to inaccessible data. We require that authors provide all relevant data within the paper, Supporting Information files, or in an acceptable, public repository. Please add a citation to support this phrase or upload the data that corresponds with these findings to a stable repository (such as Figshare or Dryad) and provide and URLs, DOIs, or accession numbers that may be used to access these data. Or, if the data are not a core part of the research being presented in your study, we ask that you remove the phrase that refers to these data.
Response: We have corrected all references so (data not shown) references have been solved.

[Note: HTML markup is below. Please do not edit.]

Reviewers' comments:

Reviewer's Responses to Questions

**Comments to the Author**

1. Is the manuscript technically sound, and do the data support the conclusions?

The manuscript must describe a technically sound piece of scientific research with data that supports the conclusions. Experiments must have been conducted rigorously, with appropriate controls, replication, and sample sizes. The conclusions must be drawn appropriately based on the data presented.

Reviewer #1: Yes

Reviewer #2: Yes

2. Has the statistical analysis been performed appropriately and rigorously?

Reviewer #1: Yes

Reviewer #2: Yes

3. Have the authors made all data underlying the findings in their manuscript fully available?

The [PLOS Data policy](http://www.plosone.org/static/policies.action#sharing) requires authors to make all data underlying the findings described in their manuscript fully available without restriction, with rare exception (please refer to the Data Availability Statement in the manuscript PDF file). The data should be provided as part of the manuscript or its supporting information, or deposited to a public repository. For example, in addition to summary statistics, the data points behind means, medians and variance measures should be available. If there are restrictions on publicly sharing data—e.g. participant privacy or use of data from a third party—those must be specified.

Reviewer #1: Yes

Reviewer #2: Yes

4. Is the manuscript presented in an intelligible fashion and written in standard English?

PLOS ONE does not copyedit accepted manuscripts, so the language in submitted articles must be clear, correct, and unambiguous. Any typographical or grammatical errors should be corrected at revision, so please note any specific errors here.

Reviewer #1: No

Reviewer #2: Yes

5. Review Comments to the Author

Please use the space provided to explain your answers to the questions above. You may also include additional comments for the author, including concerns about dual publication, research ethics, or publication ethics. (Please upload your review as an attachment if it exceeds 20,000 characters)

**Reviewer #1:**
The authors presented a study that evaluates the degradation of keratin by 4 different bacteria and the co-cultivation of these species. Interesting results are presented here, however some corrections and complements to experiments I understand can be done to improve the quality of the work.

Lines 120-121 (pg 5). Replace “The four bacterial strains used in this study were previously isolated from soil and characterized” for “The four bacterial strains used in this study were previously isolated from soil and identified by morphological analysis and 16S rRNA gene sequences.”
Response: We have edited the sentence to state that 16S rRNA gene sequence analysis was used to identify the strains. See line 125. We are not sure as to why a morphological analysis would be needed or beneficial? Is this an efficient approach across bacterial genera?

Line 126 (pg 6). “Liquid cultures were...” What was the growth conditions (for "X" hrs at ºC and rpm)?
Response: We have edited the sentence to state that; “cultures were incubated at 24°C with horizontal shaking at 250 rpm for approx. 16 hrs, and used the following day to inoculate keratin liquid medium (KLM) for keratinolytic study.” See line 131.

Line 129 (pg 6). “1%, 0.5% and 0.25% inoculum...” Please, provide the inoculum size in relative biomass (g/L: bacterial biomass/media volume). It is possible by using a standard curve: O.D. x dry biomass. The definition of inocullum is essential to reproduce the experiments!
Response: We have added a reference table for conversion of OD to cell numbers which also allow a conversion to cell numbers per unit of keratin. See S1 Fig, S1 Table and line 135-140.

Line 148 (pg 7) “min at 4°C or min. at 4°C.”
Response: We have edited the sentence to state that; “the reaction was stopped by addition of 1.4 mL 10% TCA, followed by 15 min. incubation at 4°C.” See line 159.

Line 187 (pg 8). To separate keratin there was filtration on filter paper. To separate cells from supernatant, was the material centrifuged?
Response: We have corrected the sentence to state that; “Day 2 culture supernatants were centrifuged to precipitate cells and keratin material. Thereafter proteins were precipitated from the supernatant by addition of trichloroacetic acid to a final concentration of 10%.” See line 202-204.

Line 361 (pg 15) “rhizophila in mono-culture * CFU count of X. retroflexus in co-culture” or “rhizophila in mono-culture * CFU count of S. rhizophilain co-culture”
Response: We have corrected the sentence to state that; “Expected theoretical degradation of the XS co-culture = Degradation potential of *X. retroflexus* in mono-culture per CFU * CFU count of *X. retroflexus* in XS co-culture + degradation potential of *S. rhizophila* in mono-culture per CFU * CFU count of *S. rhizophila* in the XS co-culture. For convenience a complete calculation of the XS co-culture has been added as S12 Fig.*”*
Note that as per the next comment, we have added an example of the calculation to help the reader. See line 389-393 and S12 Fig.

Lines 358-361 (pg 15) “Expected degradation for XS co-culture = degradation potential of X. retroflexus in mono culture * CFU count of X. retroflexus in co-culture + degradation potential of S. rhizophila in mono-culture * CFU count of X. retroflexus in co-culture.” The figure legend (Fig 1C and S4B) are better that this explanation. Please, provide a better information about this calculation. Use an example.
Response: We are sorry for the confusing wording. A similar concern was made by Reviewer #2, see comment 9. We have corrected the sentence to state that; “Expected theoretical degradation of the XS co-culture = Degradation potential of *X. retroflexus* in mono-culture per CFU * CFU count of *X. retroflexus* in XS co-culture + degradation potential of *S. rhizophila* in mono-culture per CFU * CFU count of *S. rhizophila* in the XS co-culture. For convenience a complete calculation of the XS co-culture has been added as S12 Fig.*”*
Note that as per the next comment, we have added an example of the calculation to help the reader. See line 389-393 and S12 Fig.

Line 405 (pg 16) Spectrophotometer? It is a colorimetric method. Please correct for “Protease and keratinase activity were measured via spectrophotometer using azocasein...”
Response: We have corrected the sentence to state that; “Protease and keratinase activity were measured on a spectrophotometer using azocasein and azokeratin dyed substrates, respectively.” See line 456-457.

Lines 414-415 (pg 17) “but not significantly higher in regards to keratinase activity...”. It is not true. The keratinolytic activity from S. rhizophila was higher than M. oxydans and P. amylolyticus, in which no enzyme activity was noted.
Response: We understand the confusion. In regards to keratinolytic activity *S. rhizophila* is nominal significantly different from both M. oxydans and P. amylolyticus. However the adjusted p-values are larger than 0.05. This is likely do to the large variance observed from *S. rhizophila* data. We have tried to the correct the sentence accordingly: ”*S. rhizophila* had the second highest proteinase and keratinase activity at day 4 (S13A and B Fig.), which was significantly higher than *M. oxydans* and *P. amylolyticus* in regards to protease activity, but only nominal significant in regards to keratinase activity (protease: 12.26 ±1.96 U/mL, keratinase: 15.57 ±4.59 U/mL, standard deviation, Lin.1).”See line 464-468.

Lines 420-422 (pg 17) “Most co-cultures had protease and keratinase activities similar to that of X.
retroflexus mono-cultures, when comparing end-point activity at day 4 for mono- and
co-cultures of X. retroflexus.” Improve. It is repetitive!
Reponse: We have tried to clarify the sentence: “At day 4, the observed protease and keratinase activities for co-cultures of X. retroflexus were similar to that of the *X. retroflexus* mono-cultures; Only the protease activity of the XS co-culture deviated significantly, and was significantly lower (p_adj_ = 0.0118, Lin.1) (Fig 3A-B).” See line 473-478.

Line 526 (pg 23) “X. retroflexus had the highest growth potential from keratin, reaching higher cell”. It does not sound well!
Response: We have tried to amend the sentence: “*X. retroflexus* and *S. rhizophila* had the highest keratin degradation potential per CFU of the four tested species. However, *X.retroflexus* was overall the species with the highest growth potential from keratin, as it was able to reach higher cell densities during cultivation.” ).” See line 597-602.

Line 531 (pg 23) “ a community-intrinsic property”. In this work, it would be interesting to check the diversity of proteases in mono and co-cultures by zimogram gel analysis and effect of inhibitors (PMSF, EDTA, IAA, N-ethylmaleimide and pepstatin A). The authors justify only analysis of proteases from X. retroflexus because the low protein concentration/bacterial growth of the other species. So, that suggested assays would be useful to evaluate the protease profile in the secretome of each mono and co-culture.
Response: This is indeed an excellent suggestion. We had a strong *a priori* on *X. retroflexus* because it was the obvious keystone species that was supporting most of the keratin degradation in our experiment. As a consequence, we have decided to prioritize and refine our proteomic analysis toward this strain for both convenience and obvious interest in its degradative potential. Nevertheless, it is indeed very true that zimograms would have provided an interesting opportunity to fingerprint and screen the three remaining species and all culture combinations with these. We have simply not considered it.

Unfortunately, as this project is now finished, these extra analyses cannot be done at the moment. However, we have added specific text in the perspectives to mention this very relevant suggestion for future research. See line 689-695.

Line 599 (pg 26) Correct “keratin degradtion”
Response: Corrected.

Line 632 (pg 27) “advantageous in an industrial setting”. Provide examples!
Response: We have added examples. See line 716-717.

**Reviewer #2:**
This manuscript presents an interesting approach on the study of biodegradation mechanisms of recalcitrant keratinous material. The article describes that co-cultures of keratinolytic strains can present synergistic collaboration to facilitate keratin degradation. Although cooperative mechanisms of microbial biodegradation have been described, similar studies on keratin are scarce and this work presents a solid experimental data to support the hypothesis. The manuscript is generally well written but needs revision for some awkward phrasing and typos.
Specific points:
1) Lines 34-35. How can you define the isolates as “well-characterized synergistic” at this point?
Response: We have corrected the sentence. We did not try to claim that these species are well-characterized for keratin degradation, as this is indeed a new avenue of investigation for this community. However, we have extensive experience working with this community for other applications, and here we consistently observe that they interact synergistically [1–4]. We therefore aimed at stating that their biofilm potential is well-characterized as synergistic. We have tried to re-formulate the sentence to make this clear to the reader. See line 33-35.

2) Line 105-106. Although complete keratin degradation is not usually achieved by individual strains, some isolates can do that. Thus, the sentence needs rewriting.
Response: We have re-written the sentence to focus more on the benefits from using mixed species communities – such as e.g. synergistic interactions. See line 107-112.

3) Several abbreviations are not defined in the Methods section. This point should be carefully revised.
Response: We have adjusted the manuscript accordingly, and all abbreviations are adjusted.

4) Line 129. This sentence is confused, please rewrite. In addition, sentences should not be initiated by numbers, please check throughout the manuscript.
Response: We have adjusted the sentence accordingly and ensure that sentences are not initiated with numbers.

5) Line 151. The source of azokeratin should be described.
Response: We have added a reference describing the procedure (Kang et al. 2018). See line 164-165.

6) Line 167. This heading is somewhat confusing. In this case, it seems that “DNA extraction from biofilm adhered to keratin particle” is sufficient.
Response: We have correct the title to a simplified version: “DNA extraction from biofilm adhering to keratin particles”. See line 181.

7) Line 192. The source and grade of trypsin should be provided.
Response: We have corrected the issue. We used Pierce^TM^ Trypsin Protease, MS Grade, from ThermoFisher Scientific

8) Results. Figures are not properly placed/labeled in the manuscript, which impairs a clear evaluation.
Response: Figure numbering and referencing has been modified and corrected upon revision of our manuscript.

9) Fig. 1. The values of “expected” keratin degradation in 1C do not match with those observed in 1A. It seems that values correspond to a normalized theoretical degradation but calculation is not clearly described here.
Response: Reviewer #1 had a similar concern; see comment to Lines 358-361. We have changed the text for better clarity and added an example of this calculation in supplementary material, see S12 Fig and S1 Table.

10) Fig. 2. Legend to this figure is somewhat unclear and should be revised. Data from Fig. 2 and Fig. S5 could be correlated with data from Fig. S1.
Response: We would like to thank the reviewer for this comment especially. Inclusion of the correlation analysis really added to the manuscript. We have corrected the figure legend, and included a full correlation analysis of most variables observed throughout the manuscript – please see comment 13 were we have compiled the output from the analysis.

11) Figure S13 could be included as Fig. 4 in the main body of this manuscript.
Response: We have added S13 Fig. as Fig. 4 to the manuscript.

12) Lines 479-480 and Table 1. Predictive function for some hypothetical proteins can be assigned by putative conserved domains tool of BLAST algorithm. The protein ID codes provided in Table 1 do not allow searching in EMBL or NCBI databases. Please provide adequate accession numbers or gi.
Response: We tried to run Delta-BLAST for the hypothetical proteins presented in Table 1 to get more information on the potential functionality of these proteins. However, when searching with Delta-BLAST the proteins matched other hypothetical proteins from the same or a related genus. Future attempts could either complement Delta-BLAST analysis with HHpred or use STRING analysis, but we would like to avoid making biological assumptions based on ‘uncertain’ annotations when we already have some meaningful annotations. We have appended the fully annotated genome (see below) with protein sequences to the manuscript so readers can decide for themselves whether to dig deeper into the predicted functions of hypothetical proteins. If needed we can include a HHpred analysis, but we fear that this will severely increase the length of the manuscript, without adding very much content. What are your thoughts on this approach??

Regarding access to the protein sequences we have been in contact with ENA-EMBL to see if it was possible to append the RAST annotated genome with protein sequences to the genome assemblies we have already uploaded (e.g. PRJEB18431 for *X. retroflexus*). Unfortunately, the response was that this feature is not currently available. ENA-EMBL estimates that it should be possible in the near future, and when the feature is available, we will added the annotated genome with protein sequences to the ENA repository. The only other possible approach would be to upload a complete replicate of the current upload with the new annotations. However, this would create unnecessary redundancy in public repositories, which we would like to avoid. As an alternative we have appended the RAST annotated genome as supplementary information to the manuscript and to the uploaded mass spectrometry data, so readers can get easy access to the genome. We are open to other suggestions.

13) Lines 575-578. This hypothesis should be reinforced correlating the observed values for proteolytic/keratinolytic activities.
Response: We would like to thank the reviewer for this valuable suggestion. Unfortunately, our current data is insufficient for solving this hypothesis, as we will try to explain with the following data presentation. Relevant figures and tables from the following analysis have been included in the manuscript as figures S14 Fig. and S15 Fig. and as tables S2 Table and S3 table. However, the analysis did yield further support for the importance of cell association to the material for enhanced degradation, as compared to planktonic cells. Note the addition of lines 425-440.

## Correlation analysis – Protease and keratinase

The acquired data on protease and keratinase activity contains 3 biological replicates for each culture type (e.g. *X. retroflexus* mono-culture [X] or *X. retroflexus* and *S. rhizophila* [XS] co-culture). Data from day 4 cultures of *X. retroflexus* mono-culture and co-cultures of *X. retroflexus* were included in the analysis. Figure 1 presents a scatter plot of protease and keratinase activity for each of the culture types with all three biological replicates included. To help visualize a potential relationship between the variables a linear regression was fitted to the data, see blue line. However, when fitting a Pearson’s or Spearman’s ranked correlation on top of the data, the correlation coefficients were very weak and not significant, see table 1 and 2.
As the effects are likely tightly linked to culture type and not necessarily linear across culture types we included a Spearman’s ranked correlation analysis.
As internal correlations within each culture type might affect the overall correlation across culture type, inclusion of all biological replicates will likely hamper the correlation analysis. We therefore tried to average the biological replicates across culture type to only include one value per culture type, see Figure 2. We complemented this approach, by performing Pearson’s and Spearman’s ranked correlation analysis on the reduced data, see table 3 and 4. The observed correlation coefficients were generally higher for the reduced data, but similar to Figure 1, no significant correlations were observed.

***Figure 1: Scatterplot of protease (Protease.Act) and keratinase (Keratinase.Act) activity for each of the culture types with all three biological replicates included. Culture type (e.g. X. retroflexus alone [X] or co-culture of X. retroflexus and S. rhizophila [XS]) is color coded according to type. Blue line represents a linear regression on the data.***

| Correlation coefficient | Statistic | P-value | Parameter | Conf.low | Conf.high | alternative |
| --- | --- | --- | --- | --- | --- | --- |
| 0.3054077 | 1.156415 | 0.2683213 | 13 | -0.2452195 | 0.7070528 | two.sided |

***Table 1: Pearson’s correlation on data presented in Figure 1.***

| Rho-estimate | Statistic | P-value | Alternative |
| --- | --- | --- | --- |
| 0.0571429 | 528 | 0.842456 | two.sided |

***Table 2: Spearman’s ranked correlation on data presented in Figure 1.***

***Figure 2: Scatterplot of protease (Protease.Act) and keratinase (Keratinase.Act) activity for each of the culture types with all three biological replicates included. Culture type (e.g. X. retroflexus alone [X] or co-culture of X. retroflexus and S. rhizophila [XS]) is color coded according to type. Blue line represents a linear regression on the data.***

| Correlation coefficient | Statistic | P-value | Parameter | Conf.low | Conf.high | Alternative |
| --- | --- | --- | --- | --- | --- | --- |
| 0.5464345 | 1.13009 | 0.3406269 | 3 | -0.6484504 | 0.9639701 | two.sided |

***Table 3: Pearson’s correlation on data presented in Figure 2.***

| Rho-estimate | Statistic | P-value | Alternative |
| --- | --- | --- | --- |
| 0.6 | 8 | 0.35 | two.sided |

***Table 4: Spearman’s ranked correlation on data presented in Figure 2.***

## Correlation analysis – All variables included

We then expanded the correlation analysis to include observations from; I) Protein concentration in the medium at day 4, II) keratin degradation in the medium at day 4, III) Total CFU counts in the medium and IV) qPCR counts of cell associated to the keratin particles (biofilm) at day 4. Biofilm observations were only included for the analysis of averaged biological replicates, as the number of biological replicates (n=6) deviated from the other data sets (n=3).

No significant correlation was observed between protease and keratinase activity for either Pearson’s or Spearman’s ranked correlations. However, a significant (p_adj_ = 0.0405) strong positive (*r* = 0.9828125) Pearson’s correlation was observed between KeratinLoss and Biofilm counts, whereas total CFU counts from the liquid culture presented non-significant (p_adj_ = 0.8) weak negative (*r* = -0.3526215) correlation to keratin degradation. Hence, the degree of cell association to the keratin particles is a good predictor for keratin degradation, supporting that biofilm formation could be linked to enhanced keratin degradation. Spearman’s ranked correlation analysis of KeratinLoss and Biofilm counts produced a strong positive correlation coefficient (*r_s_* = 1), although correlation was only nominal significant (p = 0.01667 and p_adj_ = 0.1401978).

We have added this observation to the manuscript, and supporting figures and tables have been added to supporting information. We again thank the reviewers for their insightful contribution, which have further developed the manuscript.

***Figure 3: Correlation matrix with Spearman’s ranked correlations between investigated variables. Averaged values of all biological replicates across each culture type after 4 days of cultivation was used for the correlation analysis. Protease.Act refers to measured protease activity, Keratinase.Act refers to measured keratinase activity, Protein.Conc refers to measured protein concentration in the culture supernatant, Total_CFU refers to the summed CFU counts for all species in the culture, KeratinLoss refers to amount of keratin removed in the culture during cultivation, and Biofilm refers to counts of 16S rDNA gene copies. Color intensity and circle size corresponds to the size of the correlation coefficient ranging between -1 and 1. The larger and darker blue the circle is displayed the closer the correlation coefficient is t 1. The larger and darker red the circle is displayed, the closer the correlation coefficient is to -1. Stars within the circles correspond to level of significance for the FDR corrected p-values: no star refers to P_adj_ > 0.05, * refers to 0.05 > P_adj_ < 0.01, ** refers to 0.01 > P_adj_ < 0.001 and *** refers to 0.001 > P_adj_.***

| **Pairwise comparisons** | **Cor. Coef.** | **P-value** | **P_adj_** |
| --- | --- | --- | --- |
| Protease.Act - Keratinase.Act | 0.6 | 0.2847570 | 0.4745950 |
| Protease.Act - Protein.conc | 0.7 | 0.1881204 | 0.4031152 |
| Keratinase.Act - Protein.conc | 0.9 | 0.0373861 | 0.1401978 |
| Protease.Act - KeratinLoss | 0.4 | 0.5046316 | 0.6881340 |
| Keratinase.Act - KeratinLoss | 0.8 | 0.1040880 | 0.2602201 |
| Protein.conc - KeratinLoss | 0.9 | 0.0373861 | 0.1401978 |
| Protease.Act - Total_CFU | -0.2 | 0.7470601 | 0.8619924 |
| Keratinase.Act - Total_CFU | -0.6 | 0.2847570 | 0.4745950 |
| Protein.conc - Total_CFU | -0.2 | 0.7470601 | 0.8619924 |
| KeratinLoss - Total_CFU | -0.1 | 0.8728886 | 0.8728886 |
| Protease.Act - Biofilm | 0.4 | 0.5046316 | 0.6881340 |
| Keratinase.Act - Biofilm | 0.8 | 0.1040880 | 0.2602201 |
| Protein.conc - Biofilm | 0.9 | 0.0373861 | 0.1401978 |
| KeratinLoss - Biofilm | 1.0 | 0.01667 | 0.1401978 |
| Total_CFU - Biofilm | -0.1 | 0.8728886 | 0.8728886 |

***Table 5: Spearman’s ranked correlations between all variables included in figure 3. Data analysis was performed on the mean observations across culture types and all variables. Adjusted p-values (*P_adj_) *refer to FDR corrected p-values.***

### Pearsons’s ranked correlation, summarized across biological replicates

***Figure 4: Correlation matrix with Spearman’s ranked correlations between investigated variables. Averaged values of all biological replicates across each culture type after 4 days of cultivation was used for the correlation analysis. Protease.Act refers to measured protease activity, Keratinase.Act refers to measured keratinase activity, Protein.Conc refers to measured protein concentration in the culture supernatant, Total_CFU refers to the summed CFU counts for all species in the culture, KeratinLoss refers to amount of keratin removed in the culture during cultivation, and Biofilm refers to counts of 16S rDNA gene copies. Color intensity and circle size corresponds to the size of the correlation coefficient ranging between -1 and 1. The larger and darker blue the circle is displayed the closer the correlation coefficient is t 1. The larger and darker red the circle is displayed, the closer the correlation coefficient is to -1. Stars within the circles correspond to level of significance for the FDR corrected p-values: no star refers to P_adj_ > 0.05, * refers to 0.05 > P_adj_ < 0.01, ** refers to 0.01 > P_adj_ < 0.001 and *** refers to 0.001 > P_adj_.***

| **Pairwise comparisons** | **Cor. Coef.** | **P-value** | **P_adj_** |
| --- | --- | --- | --- |
| Protease.Act - Keratinase.Act | 0.5464345 | 0.3406269 | 0.7299148 |
| Protease.Act - Protein.conc | 0.9538841 | 0.0118055 | 0.0885410 |
| Keratinase.Act - Protein.conc | 0.6067223 | 0.2779314 | 0.7299148 |
| Protease.Act - KeratinLoss | 0.1500091 | 0.8097212 | 0.8097212 |
| Keratinase.Act - KeratinLoss | 0.6566510 | 0.2286637 | 0.7299148 |
| Protein.conc - KeratinLoss | 0.4140325 | 0.4883119 | 0.7980713 |
| Protease.Act - Total_CFU | -0.2847297 | 0.6424307 | 0.7980713 |
| Keratinase.Act - Total_CFU | -0.8840038 | 0.0465902 | 0.2329512 |
| Protein.conc - Total_CFU | -0.2466145 | 0.6892132 | 0.7980713 |
| KeratinLoss - Total_CFU | -0.3526215 | 0.5605144 | 0.7980713 |
| Protease.Act - Biofilm | 0.2018748 | 0.7447217 | 0.7980713 |
| Keratinase.Act - Biofilm | 0.5653425 | 0.3206155 | 0.7299148 |
| Protein.conc - Biofilm | 0.4777624 | 0.4157008 | 0.7794391 |
| KeratinLoss - Biofilm | 0.9828125 | 0.0026979 | 0.0404691 |
| Total_CFU - Biofilm | -0.2017586 | 0.7448666 | 0.7980713 |

***Table 6: Pearson’s correlations between all variables included in figure 3. Data analysis was performed on the mean observations across culture types and all variables. Adjusted p-values (*P_adj_) *refer to FDR corrected p-values.***

14) Line 614. Could some differentially expressed proteins be associated with bacterial adhesion, aggregation or biofilm formation?
Response: Unfortunately, we did not observe any proteins associated to bacterial adhesion or similar. However, this is also expected as we are focusing on secreted proteins in the cell-free supernatant. Hence most proteins associated to adhesion and/or aggregation are probably lost during cell removal.

15) The manuscript should be thoroughly revised for typing and grammar errors.
Response: We have thoroughly revised the manuscript.

6. PLOS authors have the option to publish the peer review history of their article ([what does this mean?](https://journals.plos.org/plosone/s/editorial-and-peer-review-process#loc-peer-review-history)). If published, this will include your full peer review and any attached files.


If you choose “no”, your identity will remain anonymous but your review may still be made public.


**Do you want your identity to be public for this peer review?** For information about this choice, including consent withdrawal, please see our [Privacy Policy](https://www.plos.org/privacy-policy).

Reviewer #1: Yes: Ronivaldo Rodrigues da Silva

Reviewer #2: No


[NOTE: If reviewer comments were submitted as an attachment file, they will be attached to this email and accessible via the submission site. Please log into your account, locate the manuscript record, and check for the action link "View Attachments". If this link does not appear, there are no attachment files to be viewed.]


While revising your submission, please upload your figure files to the Preflight Analysis and Conversion Engine (PACE) digital diagnostic tool, <https://pacev2.apexcovantage.com/>. PACE helps ensure that figures meet PLOS requirements. To use PACE, you must first register as a user. Registration is free. Then, login and navigate to the UPLOAD tab, where you will find detailed instructions on how to use the tool. If you encounter any issues or have any questions when using PACE, please email us at [figures@plos.org](mailto:figures@plos.org). Please note that Supporting Information files do not need this step.

**References**

 1.	Hansen LBS, Ren D, Burmølle M, Sørensen SJ. Distinct gene expression profile of Xanthomonas retroflexus engaged in synergistic multispecies biofilm formation. ISME J. 2016; 1–4. doi:10.1038/ismej.2016.107

2.	Herschend J, Damholt ZB V., Marquard AM, Svensson B, Sørensen SJ, Hägglund P, et al. A meta-proteomics approach to study the interspecies interactions affecting microbial biofilm development in a model community. Sci Rep. 2017;7: 16483. doi:10.1038/s41598-017-16633-6

3.	Herschend J, Koren K, Røder HL, Brejnrod A, Kühl M. In vitro community synergy between bacterial soil isolates can be facilitated by pH stabilisation of the environment Downloaded from http://aem.asm.org/ on November 14 , 2018 by guest Downloaded from http://aem.asm.org/ on November 14 , 2018 by guest. 2018; doi:10.1128/AEM.01450-18

4.	Liu W, Russel J, Burmølle M, Sørensen SJ, Madsen JS. Micro-scale intermixing: A requisite for stable and synergistic co-establishment in a four-species biofilm. ISME J. 2018;12: 1940–1951. doi:10.1038/s41396-018-0112-2
